# Antibacterial Activity of Two Flavans from the Stem Bark of *Embelia schimperi*

**DOI:** 10.1155/2018/5870161

**Published:** 2018-04-30

**Authors:** Babe Guyasa, Yadessa Melaku, Milkyas Endale

**Affiliations:** Applied Chemistry Program, School of Applied Natural Science, Adama Science and Technology University, P.O. Box 1888, Adama, Ethiopia

## Abstract

*Embelia schemperi* Vatke is one of the medicinal plants used traditionally for treatment of intestinal tape worm, dysmenorrheal, bacterial, and fungal infections. Phytochemical screening test of the dichloromethane/methanol (1 : 1) and methanol extracts revealed the presence of phenols, alkaloids, tannins, and flavonoids whereas terpenoids, glycoside, and phytosterols were absent. Silica gel column chromatographic separation of the methanol extract afforded 3,5,7,3′,4′-pentahydroxyflavan, named epicatechin (**1**), along with a close flavan derivative (**2**). Structures of the compounds were elucidated by spectroscopic techniques (1D and 2D NMR, FTIR, and UV-Vis). The crude extracts and isolated compounds were screened for *in vitro* antibacterial activity against strains of *Klebsiella pneumoniae*, *Escherichia coli*, *Cryptococcus neoformans*, *Shigella dysentriae*, and *Staphylococcus aureus*. Epicatechin (**1**) exhibited comparable antibacterial activity against *S. aureus* and *E. coli* (15 and 12 mm zone of inhibition, resp.) to that of the control antibiotic gentamicin, with zone of inhibition of 15 and 12 mm, respectively, at a concentration of 20 *µ*g/mL.

## 1. Introduction

Medicinal plants have been a major source of cure for human diseases since time of immemorial. As modern techniques, for example, genomics, high-throughput screening, and target-oriented drug development strategies, have not yet fulfilled the expectations that appeared promising upon their introduction, chemotherapeutics remain the cornerstone of patient management and will likely remain so for the foreseeable future. Rapid resistance development of microorganisms to existing medicines reveals the essential need for drugs with novel modes of action. Though a number of antibacterials have been reported from natural source or their derivatives, evaluation of plant-based indigenous medicines still appears as one of the promising sources of novel antibacterial leads.

Originating from its traditional use for the treatment of various infectious diseases in Africa and Asia, the interest in the genus *Embelia* has recently increased. The genus comprises 130 species, which are mainly shrubby woody climbers distributed in tropical and subtropical areas including Africa, Eastern Asia to the Pacific Islands, and Australia [1, 2]. *Embelia schemperi* Vatke is widely used in traditional medicine as antibacterial and anthelmintic agents [3]. A gum obtained from the plant is used as a warming remedy in the treatment of dysmenorrheal, fevers, chest, and skin diseases [4]. We report here the isolation, spectroscopic identification, and antibacterial evaluation of the crude extract as well as two flavans against four selected strains of microorganisms.

## 2. Experimental Section

### 2.1. General

TLC was performed using precoated aluminum-backed supported silica gel 60 F254 (0.2 mm thickness) and glass supported silica gel 60 F254 (1.0 mm thickness), respectively. Flavonoids were detected on TLC stained with the aluminum chloride (AlC_l3_) reagent in which a positive result was indicated by the observation of pink spots visualized in vanillin. Column chromatography was carried out using silica gel 60, 70–230 mesh ASTM. The ultraviolet and visible (UV-Vis) spectrum was taken on Spectroscopic Genesys™ 2PC UV-Vis scanning spectrometer. The infrared (IR) data were recorded on a PerkinElmer model FTIR spectrometer as KBr disks. ^1^H- and ^13^C-NMR data were obtained in DMSO-*d*_6_ on a Bruker Avance 400 MHz.

### 2.2. Plant Material Collection and Identification

The stem barks of *Embelia schimperi* were collected from the Oromia region, Horo Guduru Wellaga Zone in Jarte woreda, Sombo kumi kebele, which is 381 km west of Addis Ababa on December 08, 2016. The plant was identified by the botanist Shambel Alemu, National Herbarium of Ethiopia, Addis Ababa University. The barks were cut into small pieces, air-dried, and ground into a fine powder.

### 2.3. Extraction and Isolation

Air-dried stem bark powder was weighed (500 g) and extracted exhaustively with dichloromethane/methanol (1:1) for 72 h at room temperature. The marc left was further extracted with 2 L methanol soaked for 72 h at room temperature. The mixture was filtered, and the filtrate was concentrated under reduced pressure and temperature of 40°C using rotary evaporator and afforded (20.6 g) (4.21%) crude extract. Silica gel (150 g) was mixed with 1.5 L of *n*-hexane, and the slurry was used to pack the column. The crude dichloromethane/methanol (1 : 1) extract (15 g) was adsorbed on 15 g of silica gel and applied on column. Elution was carried out with increasing gradient of ethyl acetate in *n*-hexane. A total of 36 fractions were collected each concentrated under reduced pressure to dryness. Fractions that showed similar *R*_f_ values and the same characteristic color on TLC were combined. Fraction 16 afforded single spot (compound **1**) on TLC (EtoAc/*n*-hexane, 9 : 1, *R*_f_ value of 0.52, 18 mg). Fraction 20 which showed one major spot was repurified in a small column with isocratic EtoAc/*n*-hexane (7 : 3) and afforded 12 mg of compound **2**.

### 2.4. Phytochemical Screening Test

Phytochemical screening tests were carried out following literature protocol [5–8].

*Test for alkaloids*: To 0.5 g of crude extract, dilute hydrochloric acid was added and filtered. Dragendorff's reagent (a solution of potassium bismuth iodide) was added slowly to the filtrate, and formation of red precipitate confirms the presence of alkaloids.

*Test for flavonoids*: To 0.5 g portion of crude extract, 10 mL of ethyl acetate was added and heated for 3 min using steam bath. The mixture was filtered, and the filtrate (4 mL) was mixed with 1 mL of dilute ammonia solution. Formation of intense yellow color ratifies the presence of flavanoids.

*Test for saponins*: To 0.5 g of crude extract, 5 mL of distilled water was added and shaken while heating to boil. Frothing showed the presence of saponins.

*Test for phenols*: To 0.5 g of crude extract, few drops of 2% of FeCl_3_ were added and the formation of bluish green to black color indicates the presence of phenols.

*Test for tannins*: The crude extract (0.5 g) was boiled in 10 mL of water in a test tube and filtered. To the filtrate, few drops of 0.1% ferric chloride was added to give a brownish green or a blue-black color which confirms the presence of tannins.

*Test for terpenoids*: Crude extract (0.5 g) was dissolved in 5 mL of methanol, and 2 mL of the extract was treated with 1 mL of 2,4-dinitrophenyl hydrazine dissolved in 100 mL of 2M HCl. The formation of the yellow-orange color confirms the presence of terpenoids.

*Detection of phytosterols (Salkowski's test)*: Crude extract (0.5 g) was treated with a few drops of chloroform and filtered. To the filtrate, few drops of concentrated sulphuric acid was added, shaken, and allowed to stand. Appearance of the golden yellow color indicates the presence of triterpenes.

*Test for glycosides (modified Borntrager's test)*: Crude extract (0.5 g) was treated with ferric chloride solution and immersed in boiling water for about 5 minutes. The mixture was cooled and extracted with equal volumes of benzene. The benzene layer was separated and treated with ammonia solution. Formation of the rose-pink color in the ammonical layer confirms the presence of anthranol glycosides.

### 2.5. Antibacterial Testing

#### 2.5.1. Preparation of Discs Containing Extracts

The same concentrations of 20 *µ*g/mL were prepared from the extract, isolated pure compounds, and the standard. The concentration was incorporated into sterile agar-disc diffusion and dried at 37°C. The agar disc was weighed carefully for confirming exact amount of the extract and isolated pure compounds being incorporated (compared to preweighed blank discs).

#### 2.5.2. Bacterial Culture

*Escherichia coli* and *Proteus mirabilis* which were isolated from stool specimens in the clinic were identified according to routine cultural properties and biochemical tests. Four strains of each were included in the study. A few colonies from the overnight culture of Eosin Methylene Blue (EMB) agar was transferred into approximately 4-5 mL Tripticase soy broth (TSB) medium. The broth was incubated at 37°C for 3-4 hours, and the turbidity of suspension was adjusted to that of 0.5 McFarland barium sulfate standard. The standard suspension was used for both qualitative and quantitative antibacterial assays.

#### 2.5.3. Bacterial Susceptibility Testing

Standardized inoculums (20 *µ*g/mL) were introduced on to the surface of sterile agar plates, and a sterile glass spreader was used for even distribution of the inoculums. A sterile agar-disc diffusion previously soaked in a known concentration of extract or pure compound (20 *µ*g/mL per disc) was carefully placed at the centre of the labeled seeded plate. The same procedure was used for all the MRSA strains used. The plates were incubated aerobically at 37°C and examined for zones of inhibition after 24 hr. The inhibition zones were measured with a ruler and compared with the control disc (disc containing only physiological saline).

Strains of human pathogen microorganisms used in this study were as follows: three Gram-negative bacteria *Klebsiella pneumoniae, Proteus mirabilis*, and *Escherichia coli* and one Gram-positive bacteria *Staphylococcus aureus*. The bacterial stock cultures were incubated for 24 hr at 37°C on nutrient agar medium (Oromia Public Health Research Laboratory, Adama). The bacterial strains were grown in the Mueller–Hinton agar (MHA) plates at 37°C (the bacteria were grown in the nutrient broth at 37°C and maintained on nutrient agar slants at 4°C.

The agar was melted (50°C), and the microorganism cultures were then added aseptically to the agar medium at 45°C in plates and poured into sterile Petri dishes to give a solid plate. All these experiments were performed in duplicate. The plates were incubated for 24–48 hr at 37°C for bacteria. The inhibition zones produced by the plant extracts were compared with the inhibition zones produced by commercial standard antibiotics. The minimal inhibitory concentration (MIC) was applied to the methanol extract and compound that had proved to be highly effective against microorganisms by the agar-disc diffusion method. One dilution (20 *µ*g/mL) of *E. shimperi* extract, pure compound and standard drugs were prepared in methanol using nutrient agar tubes. Mueller–Hinton sterile agar plates were seeded with indicator bacterial strains (108 cfu) and allowed to stay at 37°C for 3 hr. Control experiments were carried out under similar conditions by using gentamicin for antibacterial activity as a standard drug. The zones of growth inhibition around the disks were measured after 18 to 24 hr of incubation at 37°C for bacteria. The sensitivities of the microorganism species to the plant extract and isolated pure compounds were determined by measuring the sizes of inhibitory zones (including the diameter of disk) on the agar surface around the disks, and values <6 mm were considered as not active against microorganisms [9, 10].

## 3. Results and Discussion

### 3.1. Phytochemical Screening Test Results

The phytochemical screening results showed absence of terpenoids, glycosides, and phytosterols in the methanol extracts whereas saponins, phenols, alkaloids, tannins, and flavonoids are present. Similarly, the CH_2_Cl_2_/CH_3_OH (1 : 1) extract showed the absence of terpenoids, phytosterols, and glycosides whereas saponins, alkaloids, tannins, phenols, and flavonoids are present (Table 1).

### 3.2. Characterization of Compounds

Compound **1** was obtained as a yellowish powder (melting point 173°C) isolated from MeOH extract with *R*_f_ value of 0.52 in EtoAc/*n*-hexane (9 : 1). Its UV-Vis spectrum revealed absorption maxima at 282 nm, suggesting the presence of Π to Π^∗^ transition due to the presence of aromatic ring chromophore. The IR (KBr disk) spectrum showed broad vibration at 3375 cm^−1^ attributed to hydroxyl moiety (OH), sharp absorption at 1600 cm^−1^ attributed to aromatic benzene ring, strong absorption band at 2275 cm^−1^ due to C-H stretching of saturated moiety, and absorption band at 1255 cm^−1^ due to C-O stretching.

The ^1^H-NMR *δ*_H_ (400 MHz, DMSO-*d*_6,_Table 2) spectrum revealed the presence of proton signals at *δ*_H_ 5.73 (1H, d, *J* = 1.2 Hz, H-6) and 5.89 (1H, d, *J* = 1.2 Hz, H-8) suggest the presence of two meta coupled aromatic protons that belong to a tetrasubstituted phenyl ring A. The presence of signals with ABX multiplicity pattern at *δ*_H_ 6.88 (1H, dd, *J* = 8.1, 1.2 Hz, H-6′) and *δ*_H_ 6.73 (1H, dd, *J* = 2.1, 8.1 Hz, H = 2′, 5′) suggest a trisubstituted phenyl ring B. Signals at *δ*_H_ 4.73 (1H, d, H-2, *J* = 2.2) and 4.01 (1H, m, H-3) suggest the presence of two oxygenated methines whereas signals a peak at *δ*_H_ 2.67 (1H, dd, *J* = 16.5, 4.5 Hz, H-4a) and at *δ*_H_ 2.47 (1H, dd, *J* = 16.5, 4.5 Hz, H-4b) suggest the presence of diasterotopic methylene protons at C-4. The above ^1^H-NMR pattern suggests that the compound has flavan skeleton with two aromatic protons (H-6 and 8) on ring A and three aromatic protons (H-2′, 5′, and 6′) on ring B and devoid of the carbonyl group at C-4 of ring C.

In agreement with the ^1^H-NMR, the ^13^C-NMR spectrum (Table 2) revealed a total of fifteen carbon signals. The presence of two oxygenated sp^2^ quaternary carbons was observed at *δ*_C_ 144.7 (C-3′) and *δ*_C_ 144.6 (C-4′), suggesting the vicinal substitution pattern on ring C, in agreement with the ABX multiplicity pattern, where as the methines appear at *δ*_C_ 118.5 (C-6′) and *δ*_C_ 115.1 (C-2′, 5′). The presence of two sp^2^ oxygenated quaternary carbons at *δ*_C_ 156.7 (C-5) and *δ*_C_ 156.6 (C-7) along with two upfield carbons chemical shifts at *δ*_C_ 95.3 (C-6) and *δ*_C_ 94.5 (C-8) suggest that ring A has 5,7-dioxygenated substitution pattern. The following quaternary carbons are also clearly evident from ^13^C-NMR spectrum: *δ*_C_ 99.0 (C-4a), *δ*_C_ 131.1 (C-1′), and 156.2 (C-8a). Signals at *δ*_C_ 78.4 (C-2) and *δ*_C_ 65.2 (C-3) are clearly evident due to the presence of sp^3^ oxygenated methines C-2 and C-3 of ring C. Moreover, the presence of one methylene (also supported by DEPT-135 pointing down) observed at *δ*_C_ 28.6 (C-4) is in good agreement with spectral data, and the structure of the compound has flavan skeleton.

The COSY spectrum showed a correlation between protons at *δ*_H_ 4.01 (H-3) and *δ*_H_ 4.73 (H-2) in agreement with the substitution pattern in ring C. Similarly, aromatic proton at *δ*_H_ 5.73 (H-6) showed HMBC correlations with C-5, 7, and 8 at *δ*_C_ 156.8, 156.3, and 94.5, whereas aromatic proton at *δ*_H_ 5.89 (H-8) showed HMBC correlations with C-6, 7, 8a, and 4a at *δ*_C_ 95.3, 156.3, 156.2, and 99.0 in agreement with the substitution pattern in ring A. The methylene proton at *δ*_H_ 2.67 (H-4) showed HMBC correlations with the *δ*_C_ 78.4 (C-2), 65.2 (C-3), 156.2 (8a), and 99.0 (5a) in agreement with the substitution pattern in ring C. The HMBC correlations between the proton at *δ*_H_ 6.88 (H-6′) and *δ*_H_ 6.67 (H-5′) with that of *δ*_C_ 78.4 (C-2) and two oxygenated sp^2^ vicinal quaternary carbons at *δ*_C_ 144.6 (C-4′) and 144.7 (C-3′) further support the ABX multiplicity pattern of ring B confirming the exact location of the two hydroxyl groups to be at C-3′ and 4′ positions. Thus, based on the above spectral data the structure of the compound was found to be 3,5,7,3′,4′-pentahydroxyflavan (**1**) similar to the previously identified compound epicatechin (**1**) with various pharmacological activity [11, 12].

Compound **2** was isolated as orange powder with *R*_f_ value of 0.75 in EtoAc/methanol (9 : 1). The UV-Vis spectrum showed absorption maxima at *λ*_max_ (in MeOH) at 282 nm, suggesting the presence of Π to Π^∗^ transition due to the presence of aromatic ring chromophore. The IR (KBr disk) spectrum showed broad vibration at 3254 cm^−1^ due to the presence of the hydroxyl group, sharp absorption at 2933 cm^−1^ due to the saturated group, sharp absorption at 1599 cm^−1^ attributed to the aromatic benzene ring, strong absorption band at 2275 cm^−1^ due to the saturated group C-H stretching, and strong absorption band at 1132 cm^−1^ due to C-O stretching. All ^1^H-NMR and ^13^C-NMR features of compound **2** are very close to that of compound **1** (all NMR data are summarized in Table 3) except that the ^1^H-NMR spectra shows a AA′XX′ spin pattern with two pair of doublets at *δ*_H_ 6.88 (2H, dd, *J* = 8.1, 1.2 Hz, H-2′, 6′) and 6.73 (2H, dd, *J* = 2.1, 8.1 Hz, H-3′, 5′), suggesting a 1′, 4′ disubstituted ring B. In agreement with the ^1^H-NMR pattern, the ^13^C-NMR revealed only one oxygenated sp^2^ quaternary carbon at *δ*_C_ 144.7 (C-4′), where the methines appear at 118.5 (C-2′ and 6′) and 115.1 (C-3′ and 5′), suggesting the presence of symmetry in ring B. Thus, based on the above spectral data, the structure of compound **2** was found to be 3,5,7,4′-tetrahydroxy flavan (**2**).

### 3.3. Antibacterial Activity

The antibacterial activity of the extract and isolated compounds of *E. shimperi* were examined at a concentration of 20 *μ*g/mL against four pathogenic bacterial strains: one Gram-positive *Staphylococcus aureus* and three Gram-negative *Escherichia coli*, *Klebsiella pneumoniae*, and *Proteus mirabilis*. Antibacterial potential of crude extract and isolated pure compound were assessed in terms of zone of inhibition of bacterial growth. The results of the antibacterial activities are presented in Table 4.

As shown in Table 4, the results revealed that the isolated compounds showed promising antibacterial activity against *Staphylococcus aureus*, *E. coli*, Proteus *mirabilis* and *Klebsiella pneumoniae*. Epicatechin (**1**) exhibited comparable antibacterial activity against *S. aureus* to that of gentamicin, with the zone of inhibition diameter 15 mm. This result shows that Epicatechin (**1**) and the genus *Embelia* are potential candidates for development of antibacterial drug against *Staphylococcus aureus*.

## 4. Conclusions

This work resulted in the isolation of two flavan compounds (**1**, **2**) isolated for the first time from the stem bark of *Embelia schimperi*. The structures of the compounds were characterized on the basis of spectral data (UV-Vis, ^1^H-NMR, ^13^C-NMR, DEPT-135, HMBC, HSQC, COSY, and IR) as well as in comparison with the literature report. The antibacterial test results revealed that the isolated compounds showed promising antibacterial activity against *Staphylococcus aureus*, *E. coli*, *Proteus mirabilis,* and *Klebsiella pneumoniae*. Epicatechin (**1**) exhibited comparable (15 mm zone of inhibition) antibacterial activity against *S. aureus* to that of gentamicin (15 mm zone of inhibition). Compound **2** also exhibited promising antibacterial activity against *S. aureus* and *E. coli* and 11 and 13 mm zone of inhibition, respectively, compared to that of gentamicin (15 mm zone of inhibition).

## Figures and Tables

**Table 1:** Phytochemical screening test results.

Secondary metabolites	CH_3_OH extract	CH_2_Cl_2_/CH_3_OH (1 : 1) extract
Saponins	+	+
Terpenoids	−	−
Phytosterols	−	−
Flavonoides	+	+
Alkaloides	+	+
Phenols	+	+
Glycosides	−	−
Tannins	+	+

+ indicates presence; − indicates absence.

**Table 2:** ^1^H-NMR, ^13^C-NMR, and 2D spectral data of compound **1** in DMSO-*d*_6._

Position	^1^H-NMR	^13^C-NMR	COSY	HMBC
2	4.73 (1H, s, H-2)	78.5	H2-H3	H_2_-C_1_′_,3,4_
3	4.01 (1H, m, *J* = 2.1 Hz, H-8)	65.4	H2-H3	H_3_-C_1_′_,2,4, 4a_
4	2.67 (2H, dd, *J* = 16.5, 4.5 Hz, H-4)	28.7	H3-H4	H_4_-C_2,3,4a, 5, 8a_
4a	—	99.0		
5	—	156.9		
6	5.73 (1H, d, *J* = 1.2 Hz)	95.6		H_6_-C_4a, 5,7,8_
7	—	156.7		
8	5.89 (1H, d, *J* = 1.2 Hz)	94.6		H_8_-C_6,7,8a,4a_
8a	—	156.2		
1′	—	131.1		
2′	6.67 (1H, dd, *J* = 8.1, 2.1 Hz)	115.2		
3′	—	144.6		
4′	—	144.7		
5′		115.4		
6′	6.88 (1H, dd, *J* = 8.1, 2.1 Hz)	118.4	H5′-H6′	H_6_′-C_1_′,_3_′,_4_′,_5_′

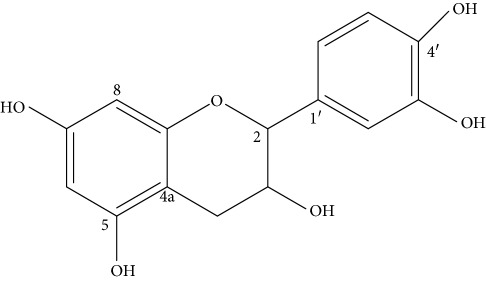				

**Table 3:** ^1^H-NMR and ^13^C-NMR spectral data of compound **2** in DMSO-*d*_6_.

Position	^1^H-NMR (ppm)	^13^C-NMR (ppm)
1′	—	131.1
6	5.89 (1H, d, *J* = 2.1 Hz, H-8)	95.3
8	5.73 (1H, d, *J* = 2.1 Hz, H-6)	94.5
2′	—	—
4a	—	99
5′	6.67 (2H, dd, *J* = 8.1 Hz, H-3′, 5′)	115.3
6′	6.88 (1H, d, *J* = 1.2 Hz, H-2′, 6′)	118.4
2	4.73 (1H, m, H-2)	78.5
3	4.01 (2H, d, *J* = 2.1 Hz, H-8)	65.2
4	2.67 (2H, d, *J* = 16.5, 4.5 Hz, H-4)	28.6
4′	—	144.7
3′	—	—
8a	—	156.2
7	—	156.5
5	—	156.8

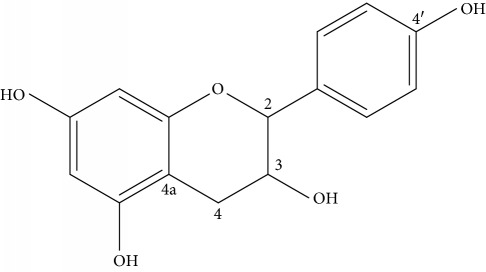		

**Table 4:** Zone of bacterial growth inhibition (mm) for crude extract and isolated compounds from the stem bark of *E. schemperi*.

Sample	*Staphylococcus aureus*	*Escherichia coli*	*Proteus mirabilis*	*Klebsiella pneumonia*
Methanol extract	*n*	*n*	*n*	*n*
Compound **2**	11	13	10	10
Compound **1**	15	12	6	11
Gentamicin	15	15	15	15

*n* ≤ 6 is null, and *n* > 6 is sensitive.
